# High-throughput sequencing of cytosine methylation in plant DNA

**DOI:** 10.1186/1746-4811-9-16

**Published:** 2013-06-07

**Authors:** Thomas J Hardcastle

**Affiliations:** 1 Department of Plant Sciences, University of Cambridge, Downing Street, Cambridge CB23EA, UK

## Abstract

Cytosine methylation is a significant and widespread regulatory factor in plant systems. Methods for the high-throughput sequencing of methylation have allowed a greatly improved characterisation of the methylome. Here we discuss currently available methods for generation and analysis of high-throughput sequencing of methylation data. We also discuss the results previously acquired through sequencing plant methylomes, and highlight remaining challenges in this field.

## Introduction

Cytosine methylation arises from the addition of a methyl group to a cytosine’s C5 carbon residue. In plant systems, cytosine methylation occurs in three sequence contexts, which have significant effects on both the mechanisms and function of methylation. The most abundant context of methylation is that which occurs within a C-G dinucleotide (CpG) [1,2], usually symmetrically on both DNA strands as maintained by the MET1 family of methyltrasferases [3]. Cytosine methylation in a non-CpG context is subdivided into the CHH and CHG contexts, where the ambiguity code H describes a non-guanine residue. The CMT3 class [4] of methyltransferases acts to maintain CHG methylation, while CHH methylation is not maintained and so is dependent on *de novo* methylation. In each context, *de novo* methylation appears to be primarily RNA-directed [3,5,6], and requires the DRM gene family. Interestingly, most CpG sites are either unmethylated or methylated in almost all cells from a single biological sample (from the same tissue), while CHH and CHG methylation shows far more variation between cells [1,2]. Demethylation of cytosines can occur either passively, through a failure of maintenance of methylation during DNA replication, or actively. Active demethylation in *Arabidopsis* depends on the ROS [7], DME [8] and DML [9] glycosylases through a base excision repair process [10]. These proteins exhibit a preference for CpG methylation but are able to act in all methylation contexts [10], and may in part be RNA-directed [11].

Methylation of cytosines in plant DNA plays a key role in the regulation of gene expression [12,13] and non-coding factors [14]. Methylation is a wide-spread and significant form of regulatory factor, with genome-wide studies in plants reporting between 5-25% [1,2,15] of cytosines as methylated. Genome wide analyses of patterns of methylation, and the ability to detect differentially methylated regions, are thus potentially of great value in a wide range of fields in plant biology, from heritable responses to environmental [16-18], biotic [19] or viral stress [20] to studies of heterosis [21] and parental specific gene expression (imprinting) [22].

In *Arabidopsis*, and other flowering plants, methylation shows strong associations with repetitive regions, small RNA producing loci, and the pericentromeric regions [12]. Methylation at these locations appears in all contexts and is primarily directed by the action of siRNAs [23,24]. The primary function of this methylation appears to be to prevent the proliferation of transposable elements [14]. Gene body methylation, in contrast, is primarily composed of CpG methylation clusters lacking non-CpG methylation [25] which associate primarily with the 3’ end of the genes. Gene body methylation has been shown to correlate with constitutively expressed genes [12] of medium to high expression [12,26]. Conversely, methylation in the promoter regions acts to repress gene expression [12].

## Sequencing the methylome

Cytosine methylation can be measured on a genome wide scale by the application of bisulphite sequencing (BS-Seq) [27]. Sodium bisulphite treatment [28] of DNA deaminates unmethylated cytosine to uracil, while leaving methylated cytosine unchanged. Amplification of these sequences then results in a thymine appearing wherever an unmethylated cytosine had existed. High-throughput sequencing of the amplified product of bisulphite treated DNA will result, in the absence of sequencing error, in a cytosine base being called wherever methylation has occured and a thymine base where no methylation is present. By aligning the sequenced reads to a reference genome, proportions of methylation can be estimated for each cytosine. Hydroxymethylation, a modification of cytosines reported in mammals [29,30] also prevents deamination by sodium bisulphite treatment [31], and so is indistinguishable from cytosine methylation in the sequenced data.

Several protocols have been suggested for reduced representation bisulphite sequencing (RRBS) [32]. These methods make use of restriction enzymes to isolate CpG-rich regions of the genome. This approach allows deeper sequencing in CpG-islands than would otherwise be possible. Moreover, knowledge of the recognition and cleavage sites of the restriction enzyme used allows increased accuracy in mapping the sequenced reads to the genome. However, these advantages must be balanced against the loss of information on much of the genome. Meissner *et al*[33], using the Bgl II endonuclease, were able to sequence only 1-2% of the mouse genome. Gu *et al*[34] outline a detailed protocol using the MspI endonuclease that likewise offers coverage of approximately 1%, though an estimated 25% of five kilobase tiling windows are expected to contain at least one sequenced read. A further drawback in using reduced representation methods is the bias that is likely to be introduced in identifying differential methylation. Differential methylation is most easily detected at a region given a high number of reads. Consequently, the discovery of differential methylation will be biased towards those regions which have the highest density of restriction sites for the enzyme used, which may not be representative of the genome as a whole. Targeted sequencing methods [35], in which specific portions of the genome are captured before bisulphite treatment and sequencing have also been suggested [36-38]. Data acquired through this approach should be relatively unbiased as the capture efficiencies should be independent of methylation status. However, the construction of probes with which to capture the desired portions of the genome will generally require a well-annotated and complete genome assembly, and adds an additional layer of complexity to the experimental design.

Enrichment-based technologies provide an alternative sequencing approach to bisulphite treatment. Methylated DNA immunoprecipitation sequencing (MeDIP-seq) [39] relies on the use of some antibody to precipitate fragments of DNA containing a methylated cytosine. Depending on the antibody used, either all methylated cytosines can be targeted, or only those in the CpG context, and methods have recently been developed for the application of the technique to low (160ng) DNA concentrations [40]. Related methods are MBD-seq [41] and MethylCap-seq [42], which use a methyl CpG binding domain protein to precipitate DNA fragments containing methylated CpG sites, with a preference for those fragments with a high density of methylated sites. As with reduced representation bisulphite sequencing, these approaches have the advantage that a greatly reduced portion of the genome need be sequenced, offering high coverage of the genome at relatively low costs. However, the identification of methylated sites is not limited to those adjacent to some restriction site, and so a genome wide view of the methylome is possible. The major drawback of these approaches is the low resolution available for identifying any methylation site, as the presence of a sequenced read implies only that at least one cytosine in the pulled down fragment is methylated; it is thus only possible to identify methylation sites to within 150-200 bases [40]. Accurate quantification of methylation levels is also problematic using these technologies as a reduction in sequenced reads may be attributed to either a decrease in methylation or a reduction in read coverage.

Before high-throughput sequencing technologies become widely accessible, similar attempts were made to measure methylation levels by applying microarray technologies to bisulphite treated genomic DNA. These required the construction of two probes for each CpG site to be analysed, one complementary to the methylated cytosine and one complementary to the unmethylated (and thus converted to uracil) cytosine. Due to the high variation in both non-specific hybridisation and hybridisation efficiencies of probes differing by even a single base, or because only probes matching the reference genome were available, controls of genomic DNA untreated by sodium bisulphite have often been used [43] to allow estimation of this variation. However, such potential sources of noise are greatly reduced or absent in high-throughput sequencing, and hence it is not usually necessary to include such controls when using this technology.

## Previous sequencing studies

Early applications of high-throughput sequencing to the *Arabidopsis* methylome allowed significant advances to be made in the characterisation of methylation patterns. Lister *et al*[2] identified substantially more cytosines displaying some degree of methylation than previously discovered, and explored the associations of methylation with small RNA abundance. Cokus *et al*[1] identified sequence motifs that associate with high and low methylation for each different context of methylation. This study also showed several periodicities in methylation, notably a ten nucleotide (the length of a helical DNA turn) period in CHH methylation.

Lister *et al*[2] and Cokus *et al*[1] also confirmed previous associations between the context of methylation and the enzymes involved in *de novo* methylation, maintenance of methylation and demethylation. Recent work by Stroud *et al*[44] has substantially extended and refined the characterisation of regulatory factors of the methylome by examining eighty-six *Arabidopsis* mutants, suggesting that individual sites of methylation may be regulated by novel RNA-directed pathways in addition to identifying new components of known pathways. However, some care must be taken in interpreting the methylomes identified in knock-out studies, as illustrated by Havecker *et al*[45], in which a region exhibiting differential methylation between a wild-type strain of *Arabidopsis* and an *ago5* knockout was identified as a spontaneous and heritable change in methylation rather than one dependent on the AGO5 protein. The work of Schmitz *et al*[46] and Becker *et al*[47] in *Arabidopsis thaliana* examined such events on a genome-wide scale, showing that, over several generations, genetically identical individuals under controlled environmental conditions acquire variation in methylation status at numerous locations. The presence of such metastable changes in methylation status independent of genomic variation has also been observed in two inbred lines of maize [48].

Characterisation of genome-wide patterns of methylation in plant systems have largely been carried out in the model organism *Arabidopsis*. However, high-throughput sequencing technologies make the analysis of a methylome in any organism with a reference genome relatively straightforward. Feng *et al*[49] carried out shotgun sequencing of methylation in the flowering plants rice, poplar and *Arabidopsis* in a study comparing plant and animal methylomes. Zemach *et al*[15] carried out a similar study in which the methylomes of rice and *Arabidopsis* were sequenced. Distributions and abundances of methylation in each sequence context appear broadly similar in the flowering plants across gene regions, exon/intron boundaries, and repetitive regions, suggesting that the mechanisms involved in methylation identified in *Arabidopsis* are conserved in other flowering plants. More distant species appear to show substantial divergence in methylation profiles. The early diverging land plants *Selaginella moellendorffii* and *Physcomitrella patens* show almost no gene body methylation in any sequence context, although the pattern of methylation is similar to that in flowering plants around repeat regions [15]. The green algae *Chlorella sp.* NC64A and *Volvox carteri* show very little methylation in non-CpG contexts in genes, and greatly reduced or absent non-CpG methylation at repetitive regions, with *Volvox carteri* showing greatly reduced methylation in all contexts compared to other plant species [15]. Similarly, the distributions of methylation in the green algae *Chlamydomonas*, while not wholly divergent from those in flowering plants [49], show much lower levels of methylation at both genes and repetitive regions than *Arabidopsis*. Moreover, the relationships between CpG and non-CpG methylation differ substantially in *Chlamydomonas* from those in flowering plants, suggesting that the mechanisms involved have diverged, as previously reported [50].

## Alignment

The first step in analysis of high-throughput sequencing data specific to BS-Seq is that of alignment. Multiple alignment tools have been developed for the alignment of bisulphite treated sequence data. Perhaps surprisingly, these can show substantial differences in performance and quality of mapping [51,52], considerations which appear to depend chiefly on the underlying alignment algorithm used. Several BS-seq aligners make use of existing alignment tools, notably Bowtie [53-57] and SOAP package [58], both methods exploiting Burrows-Wheeler transformations [59] for rapid low-memory alignments. Alignment methods based upon customised hashtable matching [60-62], adaptive seeding and Blast-like alignment [63] have also been developed specifically for BS-Seq data. As is usual in alignment of high-throughput sequencing data, the trade-offs are principally those of computational time against the total number of reads for which an alignment is found.

The alignment of BS-Seq data does differ in one key respect from alignment of ordinary sequence data in that the conversion of unmethylated cytosines to uracil (sequenced as thymine) decreases the total information available for alignment of the sequenced reads against a reference genome. Two conceptual approaches have been suggested to address this issue. The first approach is to align sequenced cytosines to reference genome cytosines, and to align sequenced thymines to either cytosines or thymines in the reference genome. The BSMap [58] and RMAP-BS [64] aligners take this approach. The alternative approach removes the bias towards alignment of methylated reads at the cost of degrading the total information available for alignment. An *in silico* conversion of each cytosine to thymine is carried out on both the sequenced reads and the reference genome and alignment carried out on these data. Having constructed an alignment from these data, the original sequence information can then be used to call the presence or absence of methylation at each cytosine location. Most methods for aligning bisulphite sequence data (e.g. BS-Seeker [54], Bismark [55], MethylCoder [56], BRAT [60]) take this approach.

**Figure 1:**
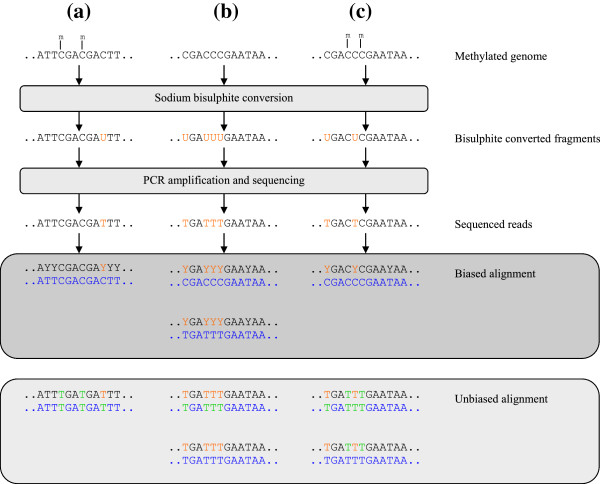
**Alignment choices for bisulphite treated data.** Biased and unbiased alignments of bisulphite treated data. Bisulphite treatment converts unmethylated cytosines to uracil, which are sequenced as thymine. In a biased alignment, sequenced thymines are treated as ambiguously cytosine/thymine (ambiguity code Y). In case (**b**), of a unmethylated read, this ambiguity allows the converted read to align to two separate locations on the reference genome (blue), while in case (**c**), which consists of the same nucleotide sequence but contains methylated cytosines, the read aligns to a single location. This results in a greater confidence in the alignment of the methylated read. In the case of an unbiased alignment, all cytosines on both the sequenced reads and the reference genome are converted to thymines, and the sequences in both (**b**) and (**c**) align to the same locations on the genome, with no additional confidence in the alignment of the methylated read. In case (**a**), the read aligns to a single location in both a biased and unbiased alignment; however, in the unbiased case less information is available to make this alignment.

A biased alignment of the sequenced reads makes maximum use of the available information and should allow the successful and unambiguous alignment of the largest possible number of sequenced reads. However, this approach is biased towards an alignment of the methylated reads. Sequenced reads from unmethylated locations contain less information after bisulphite conversion and are thus more difficult to align than sequenced reads from methylated locations. Conversely, an unbiased alignment, by converting *in silico* all sequenced cytosines to thymines ensures that the methylated reads used for alignment contain no more information than the unmethylated reads, and removes this bias. However, with less information available, fewer reads will align unambiguously to the genome. Figure 1 illustrates this distinction.

The use of SOLiD [65] technology to identify those cytosines that have undergone conversion to thymine as a result of bisulphite technology has been attempted [66]. This technology, in which overlapping di-nucleotides rather than single bases are encoded in ‘colourspace’, allows robust detection of single nucleotide mismatches between sequence and reference. This appears attractive for methylation analysis, in which unmethylated cytosines will result in such mismatches; however, many reads contain too many mismatches to be aligned with standard tools for such data. Moreover, the encoding of di-nucleotides by this technology means that a single measurement error in a read results in all downstream bases of that read being incorrectly identified, and so any attempt to call individual bases of reads and then align using standard tools will be error-prone. This also prevents the straightforward use of *in silico* conversion of cytosines to thymines, and hence an unbiased mapping of reads. Methods for unbiased mapping must instead consider each possible subsequence of the genome within colourspace [67] that might arise from the presence of either a cytosine or thymine at the genomic positions originally containing a cytosine. Such an approach is necessarily highly computationally intensive, and attempts have been made to reduce the computational load by filtering on likely methylation patterns [68] or by assuming relatively low numbers of mismatches between the sequenced reads and reference genome [69].

## Post-alignment analysis

Following alignment, analysis of methylation data can proceed upon two main paths. The first path, which has been that predominantly considered in studies to date, is the discovery of genome-wide associations of methylation, on either an annotation [2,15,49] or sequence [1] level. Analyses of this type are attractive as each methylated site (perhaps within a particular context, or in proximity with some known annotation feature) may be considered a replicate case. The need for sequencing biological replicates is thus largely removed.

The second form of analysis is that which attempts to identify individual methylation sites or loci that exhibit some behaviour of interest, usually differential methylation. Analyses of this type have been suggested by the work of Schmitz *et al*[46] and Becker *et al*[47], however, these studies limited themselves primarily to identification of genome-wide associations of differentially methylated regions rather than analysis of individual sites.

Analysis of individual locations of the methylome requires the evaluation of the methylation status of each cytosine within each sequenced biological sample. The methylation status of a given cytosine is not necessarily preserved across multiple cells in the same biological sample. For each cytosine on the reference genome the number of reads which identify that cytosine as methylated and the number of reads which identify that cytosine as unmethylated can be identified. This pair of values will in most cases form the basis of subsequent analysis of the methylome. However, several sources of variation exist that complicate the analyses of these data.

**Figure 2:**
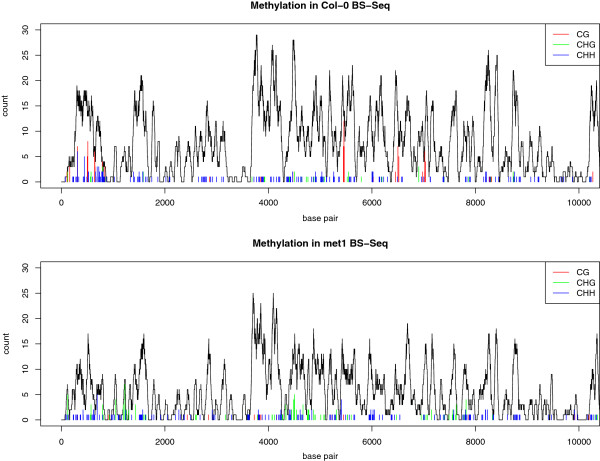
**Post-alignment data.** An example of the data available for analysis of methylation following alignment of the sequenced reads to a reference genome. The data are taken from Lister *et al*[2] and show the first ten thousand bases of the positive strand of two samples of *Arabidopsis* in wild type (Col-0) and *met1* knockout mutant. The number of times a cytosine is observed to be methylated is shown by the height of the coloured bars, with the colour indicating the context of methylation. The abundance of reported methylation is heavily dependent on the read coverage (black curve) at each base, which exhibits high variability.

A large source of variation in the number of methylated cytosines counted at a given site is the read coverage at that location. Figure 2, a re-analysis of BS-Seq data (GEO series GSE10966) from wild-type (Col-0) and *met1* mutant in the Lister *et al* study [2] demonstrates the signficance of coverage on the abundance of methylated sites, as in many locations the reported coverage drops to very low levels and makes reliable identification of methylation or differential methylation highly problematic. In this instance, the variation in coverage appears to be conserved between the two sequencing runs shown, suggesting a bias in sample preparation or alignment. Such variation is the principal reason that BS-Seq data must be considered not simply as a count of the number of methylated cytosines observed at a given location, but as a pair of values describing the both the number of methylated and unmethylated cytosines at that location.

The base calling reported by the sequencing technology is likely to contain errors [70,71], with an average 0.16% base substitution rate being reported in Illumina HiSeq data. However, this error rate may be substantially higher (up to 8.83% has been reported) at specific genomic locations, and is generally higher in GC-rich regions [70]. This may lead to either a cytosine being incorrectly reported where a thymine is present, or a thymine being incorrectly reported where a cytosine is present. In either case, the alignment of the read will not be affected, but the methylation status of that base will be incorrectly reported. Incorrect calling of other bases may also lead to errors in alignment, as may differences between the genome of the sequenced samples and the reference genome. Where a read aligns incorrectly, any sequenced cytosines and thymines which by chance align to a cytosine in the reference genome will result in an incorrect evaluation of methylation at that location.

A further source of noise in high-throughput sequencing of bisulphite treated DNA is the incomplete conversion of unmethylated cytosines to uracil. Where this occurs, a cytosine rather than a thymine will be sequenced, and will therefore be treated as evidence of methylation at that base. For a given sample, the rate of incomplete conversion may be estimated by considering those sequenced reads mapping to the chloroplast genome [2], which appears to be generally unmethylated [72]. In the Lister *et al* study, incomplete conversion rates were estimated at between 1-3

In addition to the technical sources of noise described above, the identification of consistent differential methylation must also account for biological variation in methylation status. At present, few studies are available with which to determine the variability of methylation status between biological replicates. Hansen *et al*[57] showed in a study of human cancers that there exist regions of the genome that show substantial variation in the prevalence of methylation between individuals. Such variation is perhaps to be expected in oncological studies, which tend to be heterogeneous in many respects, and is likely to be reduced in biological replicates under more stringent control of environmental conditions. However, the spontaneous changes in methylation identified between individuals separated by a relatively small number of generations [46,47] suggest that some level of variability will exist in almost all circumstances.

Proper use of biological replicates can be used to control for both technical noise and biological variation within sequencing data. In order to effectively use the data from biological replicates, properly designed statistical tools are required. Several methods have been proposed for methylation analysis of microarray data [73], however, the properties of high-throughput sequencing data are sufficiently different to those of microarray data that new analytical tools are required. These tools should account for both the characteristics of the data describing the methylation status of a single cytosine and the ‘large *n*, small *p*’ nature of high-throughput sequencing data. Methods that account for biological replication and the dimensionality of the data have been developed for the analysis of data produced from RNA-Seq experiments [74-76] that ‘borrow’ information from the whole dataset in order to improve power when evaluating individual data points. However, the data acquired for the methylome is qualitatively different from that produced by RNA-Seq experiments in that, for each sample, the information for a methylation region is defined by a pair of numbers; the number of unmethylated cytosines and the number of methylated cytosines sequenced from that region. Generalised linear model approaches have been suggested for the analysis of paired data from high-throughput sequencing [77], and these may be appropriate to the analysis of methylation data. Our recently developed approach for the detection of differential expression in paired data [78] is also suitable for the discovery of differential methylation from biological replicates.

## Conclusions

The application of high-throughput sequencing to evaluation of cytosine methylation has already made significant contributions to characterisation of the functions and mechanisms of methylation in plant systems. Bisulphite sequencing is the current gold standard for genome wide mapping of the methylome, offering largely unbiased, base-level resolution maps of methylation. Alternative methods, while usually offering higher coverage over some regions of the genome, either exhibit strong biases in the portions of the genome sequenced, or are of low resolution. Plummeting sequencing costs [79] suggest that the advantages offered by these methods over bisulphite sequencing are unlikely to outweigh these drawbacks.

Despite the progress being made in analysis of high-throughput sequencing data and specifically that pertaining to the methylome, there exist some clear areas for improvement. The question of *multireads*[80], sequenced reads that map equally well to multiple locations on the genome has yet to be comprehensively addressed. Most currently existing analyses simply discard multireads in constructing genome-wide maps of the methylome [2,46,47] and this strategy is implemented without alternatives in most alignment tools. Given the known association of methylation with repetitive elements [12], this may be a suboptimal strategy, as reads from these regions will be more likely to match to multiple locations and consequently be discarded. The development of tools for the methylome such as RSEM [81] or IsoformEx [82], which attempt to resolve the location of multiply mapping reads in RNA-Seq analyses by considering the signal observed in the uniquely mapping reads, appears an attractive strategy. However, several problems make this a substantially more challenging problem in the methylome. There is likely to be considerably more variation within the methylation status of neighbouring cytosines than exists in the reads sequenced from an RNA transcript, and so the inferences that can be made from uniquely mapping reads are less reliable. Computational difficulties also arise due to the loss of information introduced by cytosine to thymine conversions; as a consequence of this, it is possible for a converted read to map to multiple locations but carry different information at each location, depending on which sequenced thymines map to thymines and which to cytosines at each location.

A further challenge in analysis is the robust identification of methylation ‘loci’; regions of the genome where neighbouring cytosines have a positively correlated methylation status that implies co-regulation. This has been partially addressed through moving windows [2], by merging any neighbouring cytosine positions within fifty bases of each other showing similar patterns of methylation [47], and by identifying bins of some minimal length that contain some minimal number of methylated cytosines before merging those bins that are sufficiently close [44,46]. These approaches give an initial approximation to methylation loci; however, since the loci are defined based on arbitrarily defined thresholds the results may vary not only with these parameters but the depth of sequencing and the extent to which sources of noise, especially incomplete bisulphite conversion, are present within the data. Dependent on the system being studied, variation in methylation between biological replicates may also need to be accounted for when defining methylation loci.

Most genome wide analyses of methylation have been carried out in *Arabidopsis*, as this model organism possesses a small, well annotated genome with relatively few repetitive regions, however, comparisons with distributions in methylation in other flowering plants [15,49] suggest that many mechanisms are conserved. With both the generation and analysis of bisulphite sequencing data becoming increasingly straightforward, characterisation of the methylome of a diverse range of species, including non-model and crop plants, is likely to take place in the near future.

Although a wide range of tools have been developed for alignment and quantification of methylation levels from bisulphite sequencing data, methods for the post-alignment analysis of quantified methylation levels are less well developed at present. Analyses have thus far focused primarily on characterisation of genome-wide properties of methylation and, as such, have identified multiple significant factors influencing the presence or absence of methylation. A largely unaddressed problem thus far has been the identification of individual regions of differential methylation, allowing the integration of the methylome into a systems biology framework to be assessed. Appropriate methods for such analyses are now becoming available, and large scale studies of the regulatory effects of the methylome are likely to follow.

## Competing interests

The authors declare that they have no competing interests.
